# An Unsupervised Detection-to-Mitigation Framework for Resource Exhaustion Attacks in 5G/6G Network Slicing

**DOI:** 10.3390/s26123777

**Published:** 2026-06-13

**Authors:** Ja-Eun Kim, Hye-Yoon Jeong, Jae-Hyun Pi, Myung-Sun Baek, Hyoung-Kyu Song

**Affiliations:** 1 Department of Information and Communication Engineering, Sejong University, Seoul 05006, Republic of Korea; thinkdana@sju.ac.kr (J.-E.K.); zalfenz@sju.ac.kr (H.-Y.J.); 2 Department of Convergence Engineering for Intelligent Drone, Sejong University, Seoul 05006, Republic of Korea; jaehyun01@sju.ac.kr; 3 Department of AI Convergence Electronic Engineering, Sejong University, Seoul 05006, Republic of Korea; 4 Department of Artificial Intelligence and Information Technology, Sejong University, Seoul 05006, Republic of Korea; msbaek@sejong.ac.kr

**Keywords:** IoT sensor networks, mMTC slice, network slicing security, resource exhaustion attack, slice-level service availability, detection-to-mitigation framework, allocation-aware mitigation, unsupervised anomaly detection, 5G/6G networks

## Abstract

Massive Internet of Things (IoT) and sensor-network services in 5G/6G systems increasingly rely on network slicing to support large-scale sensing, monitoring, and mission-critical applications. In such sliced infrastructures, Proportional Fair (PF) allocation assigns resources according to slice-reported demands. This reliance on trusted demand reporting makes coexisting slices, including mMTC-based IoT sensor slices, vulnerable to resource exhaustion attacks, where a malicious slice inflates its demand to monopolize shared resources and induce Service Level Agreement (SLA) violations. Existing unsupervised defenses mainly focus on anomaly detection, while the translation of detection results into resource-level mitigation remains insufficiently addressed. To bridge this gap, this paper proposes AutoGuard-Hybrid, an unsupervised detection-to-mitigation framework that combines complementary anomaly detectors with allocation-aware mitigation policies to preserve slice-level service availability. Unlike prior detection-only approaches, AutoGuard-Hybrid converts unsupervised anomaly evidence into allocation-aware demand purification before PF scheduling. Its key design is a closed-loop integration of Isolation Forest (IF) and Long Short-Term Memory Autoencoder (LSTM-AE) as spatial and temporal front-end detectors with Adaptive Clipping and a Safety Cap, which translate anomaly scores into demand purification actions. Experiments show that AutoGuard-Hybrid remains comparable to Isolation Forest under Continuous attacks and improves the mean system-wide SLA violation rate by 27.6% under Adaptive Probing attacks. Stage activation analysis further shows that LSTM-AE activations increase from 9.3 under Continuous attacks to 29.4 under Adaptive Probing attacks. Ablation results show that Adaptive Clipping alone reduces the system-wide SLA violation rate by 75.0%, while the full mitigation pipeline achieves an 84.6% total reduction. AutoGuard-Hybrid operates within the 1 ms Transmission Time Interval (TTI) constraint and provides a practical defense framework for next-generation network slicing-enabled IoT and sensor-network services.

## 1. Introduction

### 1.1. Background

The advent of fifth-generation (5G) and emerging sixth-generation (6G) wireless networks has transformed the landscape of mobile communications and has accelerated the deployment of large-scale Internet-of-Things (IoT) and sensor-network services [1,2]. In particular, smart infrastructure, industrial monitoring, environmental sensing, and disaster surveillance applications require the reliable delivery of massive sensing data under heterogeneous throughput, latency, reliability, and connectivity requirements. To support such diverse services over a shared physical infrastructure, a flexible resource management paradigm has become essential [3,4]. A defining technological pillar of these architectures is network slicing, which enables a single physical infrastructure to be partitioned into multiple isolated logical networks, each tailored to specific service requirements [5]. The 3GPP standard defines three canonical slice types: enhanced Mobile Broadband (eMBB) for high-throughput multimedia applications, Ultra-Reliable Low-Latency Communications (URLLC) for mission-critical services such as autonomous driving and remote surgery, and massive Machine-Type Communications (mMTC) for large-scale IoT and sensor-device deployments. Among the various scheduling algorithms employed for resource allocation, the Proportional Fair (PF) policy has been widely adopted because it balances throughput and fairness across heterogeneous slices [6,7]. In demand-driven slicing, the PF allocation weight of slice *i* can be expressed as being proportional to $p_{i}d_{i}^{\mathrm{rep}}\left(t\right)$, where $p_{i}$ is the slice priority and $d_{i}^{\mathrm{rep}}\left(t\right)$ is the reported demand. Since the final allocation is normalized by the aggregate weighted demand of all slices, even a small perturbation in one slice’s reported demand changes both its own allocation share and the remaining shares assigned to coexisting slices. Therefore, the behavior of the PF allocator is tightly coupled to the integrity of slice demand reports.

This dependence on reported demand is a natural design choice in network slicing, where multiple tenants, including IoT sensor-service providers, share common physical resources and the allocator relies on demand reports for fair distribution [3,4]. Under this model, however, an authorized but compromised slice can inflate its reported demand to obtain a disproportionate share of the resource pool, leading to resource exhaustion attacks [8,9]. Such attacks deprive legitimate slices of their allocated resources and induce cascading Service Level Agreement (SLA) violations across the network. The consequences are particularly severe not only for URLLC slices, where even brief disruptions can compromise safety-critical applications, but also for mMTC-based IoT sensor slices, where resource starvation may delay or suppress large-scale sensing reports [1,2]. As 6G networks evolve toward fully softwarized and programmable architectures, the surface for slice-internal manipulation is expected to expand, making robust slice-level defense mechanisms an important research priority [8,10].

Defending against resource exhaustion attacks is challenging for three intrinsic reasons. First, the attacker resides within an authorized slice, so perimeter-based security models are not effective. Second, attack patterns can range from blunt continuous flooding to Adaptive Probing strategies that dynamically evade detection. Third, in real deployments, labeled attack data is rarely available, especially for newly emerging IoT and sensor-network attack scenarios. Therefore, unsupervised defense approaches that generalize to novel attack vectors are required [11,12,13]. These constraints motivate the need for an unsupervised defense framework that not only detects heterogeneous attack patterns but also translates detection evidence into adaptive resource-level mitigation actions to preserve the availability of coexisting sensor, IoT, and mission-critical slices.

### 1.2. Related Work

Recent studies on 5G/6G network slicing security have established the general security landscape of virtualized and multi-tenant slicing environments. Comprehensive surveys have summarized slicing-specific attacks, security challenges, mitigation approaches, and open research directions [8]. Practical threat analyses have further highlighted risks arising from shared slice infrastructures, including slice isolation failures, orchestration vulnerabilities, and cross-slice attack surfaces [10]. Alongside these threat analyses, machine learning-based security approaches have been investigated to improve threat detection and adaptive protection in 5G slicing systems [14], while isolation-oriented mechanisms have been studied to mitigate DDoS attacks on 5G core network slices [15]. These studies show that improper slice management can compromise confidentiality, integrity, and availability, and may even deny service to prioritized users. In sliced IoT and sensor-network environments, such availability degradation can directly affect sensing continuity, monitoring reliability, and timely data delivery.

Despite this broad security coverage, existing slicing-security studies have paid relatively less attention to slice-internal resource manipulation, where an authorized slice abuses legitimate reporting interfaces to obtain an unfair share of common resources. Recent work has examined adaptive shared-slice security using deep learning-based cyber-threat analysis [16], cross-layer slice protection based on SDN, NFV, and AI mechanisms [17], and federated learning-based architectures for intelligent native slicing security [18]. Although these studies strengthen broader slice security and cyber-threat detection, they do not directly address demand-report manipulation against proportional resource allocators. Unlike external intrusion or inter-slice isolation failures, this threat originates from within a valid slice and therefore cannot be effectively addressed by conventional access control or perimeter-based security mechanisms. In particular, resource exhaustion attacks against demand-driven allocators exploit the proportional nature of resource distribution, causing cascading SLA violations without necessarily violating authentication or slice isolation policies. This threat is especially relevant to shared infrastructures that host mMTC slices because large numbers of IoT sensor devices may depend on stable resource availability for periodic or event-driven sensing reports.

To detect such abnormal slice behaviors, recent defense approaches have increasingly adopted unsupervised anomaly detection techniques, including statistical thresholding, tree-based isolation methods, kernel-based boundary estimation, and sequential reconstruction models. Statistical anomaly detection provides a lightweight baseline for identifying deviations from normal traffic profiles, but it often relies on restrictive distributional assumptions and may be sensitive to bursty traffic variations [12]. Isolation Forest detects spatial outliers by isolating anomalous samples with shorter path lengths, making it effective for high-magnitude demand inflation, but it treats each observation independently and does not explicitly model temporal dependencies [19]. One-Class SVM estimates the support of normal data through a kernel-based boundary, but its performance depends strongly on kernel and hyperparameter choices, which can limit robustness in dynamic slicing environments [20]. Sequential reconstruction models such as LSTM-Autoencoders capture temporal deviations through reconstruction errors and are useful for gradual demand manipulation, but they may suffer from false positives during legitimate traffic bursts and provide weaker discrimination for obvious spatial anomalies [13,21]. These method-specific limitations show that existing unsupervised detectors provide useful anomaly evidence, but they do not by themselves specify how abnormal demand reports should be modified before PF scheduling.

Beyond conventional anomaly detection, adaptive learning mechanisms have also been investigated for dynamically changing environments. Cyclic reinforcement learning has been used to capture time-varying learner preferences and generate personalized tutorials in autonomous online learning environments [22]. DRL-based coding adaptation has also been applied to resource-constrained underwater acoustic sensor networks, where decisions must be adjusted under channel dynamics and limited feedback [23]. These studies suggest that adaptive learning can be useful when system behavior changes over time and feedback is limited or delayed. However, they focus on different application domains and do not address slice-internal demand manipulation or the translation of anomaly evidence into resource-level mitigation in 5G/6G network slicing.

These limitations highlight a remaining gap between unsupervised anomaly detection and resource-level mitigation in demand-driven network slicing. General anomaly detection studies provide broad foundations for identifying abnormal patterns, but they do not define how detected abnormal demands should be handled in a resource allocation loop [12]. Recent inter-slice anomaly detection methods improve the detection of distributed slice-related attacks, but their primary objective remains anomaly identification rather than allocation-aware demand purification before scheduling [13]. In particular, existing approaches often treat anomaly detection as an isolated classification task, leaving open the question of how detected abnormal demand reports should be modified before PF scheduling to preserve system-level SLA integrity. For IoT sensor slices, this gap is critical because detection alone cannot prevent sensing-service degradation unless suspicious demand reports are actively purified before resource allocation.

### 1.3. Contributions

To address this gap, this paper proposes AutoGuard-Hybrid, an unsupervised detection-to-mitigation framework for resource exhaustion attacks in 5G/6G network slicing environments that support IoT and sensor-network services. The framework combines complementary unsupervised detectors with allocation-aware mitigation policies, translating spatial and temporal anomaly evidence into demand purification actions before resource scheduling. By integrating Isolation Forest and LSTM-AE with adaptive resource-control mechanisms, AutoGuard-Hybrid aims to preserve SLA integrity and service availability for coexisting eMBB, URLLC, and mMTC slices. The main contribution of this work is not the individual use of Isolation Forest, LSTM-AE, or threshold-based clipping but their integration into a PF-aware closed-loop framework that transforms unsupervised anomaly scores into demand-level mitigation actions. This design explicitly addresses the gap between detecting abnormal slice behavior and preventing the detected abnormal demand from distorting proportional resource allocation. The specific contributions are summarized as follows.

1. **Resource-Allocation-Aware Threat Modeling.** Resource exhaustion attacks against the PF allocator are formulated as an adversarial demand manipulation problem, where a compromised but authorized slice inflates its reported demand to distort proportional resource allocation. Two attack regimes are considered: Continuous attacks with high-magnitude spatial outliers and Adaptive Probing attacks that adjust amplification based on allocation feedback. The threat model is evaluated in a representative eMBB-URLLC-mMTC slicing scenario, where the mMTC slice represents large-scale IoT sensor traffic requiring stable resource availability.
2. **Unsupervised Detection-to-Mitigation Framework.** A resource-control framework is developed to convert unsupervised anomaly scores into adaptive demand purification actions. Instead of treating detection as an isolated binary classification task, the framework modifies suspicious demand reports before PF scheduling to support SLA-preserving resource allocation for heterogeneous slices, including IoT sensor slices.
3. **Regime-Aware Detection Front-End.** A two-stage detection front-end provides complementary anomaly evidence for mitigation. Isolation Forest captures spatially evident abnormal demands, while LSTM-AE identifies temporally distributed deviations that may remain within normal marginal ranges.
4. **Adaptive Demand Purification Policy.** An adaptive purification policy composed of Adaptive Clipping and a Safety Cap is introduced. Adaptive Clipping performs score-proportional demand reduction, while the Safety Cap prevents extreme resource monopolization by demoting excessive reports to normal operating levels. This policy prevents a compromised slice from starving coexisting slices that carry mission-critical or massive IoT sensing traffic.
5. **Experimental Validation.** AutoGuard-Hybrid is evaluated under Continuous and Adaptive Probing attacks against representative unsupervised baselines. Results show that it matches the strongest single-method baseline under Continuous attacks and achieves a 27.6% improvement in the mean system-wide SLA violation rate under Adaptive Probing attacks. Ablation and stage activation analyses further confirm the role of adaptive mitigation in system-level resilience and slice-level service availability.

The remainder of this paper is organized as follows. Section 2 presents the system and threat models. Section 3 describes AutoGuard-Hybrid. Section 4 evaluates its performance, and Section 5 concludes the paper.

## 2. System Model

To improve readability, the main notation used in the system model, threat model, and proposed framework is summarized in Table 1.

### 2.1. Network Slicing and Resource Allocation Model

This study considers a network slicing environment in a 5G/6G base station (gNB) where a single physical network is partitioned into multiple logical networks [24,25]. The set of slices is defined as N={1,2,3}, corresponding to eMBB (Slice 1, the adversarial slice), URLLC (Slice 2), and mMTC (Slice 3), respectively. The system has a total normalized resource capacity of $R_{\mathrm{total}}=100$, and time progresses in discrete steps t∈{0,1,…,T−1} with T=200, where each step approximates one Transmission Time Interval (TTI) in 5G systems.

The true traffic demand of slice i∈N at time *t* combines a stochastic base demand $d_{i}^{\mathrm{base}}\left(t\right)∼N\left(\mu_{i},\sigma_{i}^{2}\right)$ and a Bernoulli-triggered burst multiplier $B_{i}\left(t\right)$:

(1) $$d_{i}^{\mathrm{true}}\left(t\right)=d_{i}^{\mathrm{base}}\left(t\right)\cdot B_{i}\left(t\right),\;B_{i}\left(t\right)=\left\{\begin{array}{cc} M_{i}^{\mathrm{burst}}, & w.p.\;P_{i}^{\mathrm{burst}}\\ 1, & w.p.\;1-P_{i}^{\mathrm{burst}} \end{array}\right..$$

The observation collected by the gNB further incorporates measurement noise:

(2) $$d_{i}^{\mathrm{obs}}\left(t\right)=d_{i}^{\mathrm{rep}}\left(t\right)\cdot \left(1+\epsilon \right),\;\epsilon ∼N\left(0,\sigma_{\mathrm{obs}}^{2}\right),$$

where $\sigma_{\mathrm{obs}}=0.02$ and $d_{i}^{\mathrm{rep}}\left(t\right)$ is the demand reported by slice *i* to the gNB. For legitimate slices, $d_{i}^{\mathrm{rep}}\left(t\right)=d_{i}^{\mathrm{true}}\left(t\right)$, while adversarial slices may manipulate this value as defined in Section 2.2. The slice-specific parameters, derived from 3GPP TS 23.501 specifications [25,26], are summarized in Table 2.

Resource distribution among slices follows the Proportional Fair (PF) scheduling policy [25,27]. The PF allocator is formulated as a convex optimization problem that maximizes the total logarithmic utility:

(3) $$\max_{x(t)}\sum_{i\in N}p_{i}d_{i}^{\mathrm{rep}}\left(t\right)\log\left(x_{i}\left(t\right)\right)\;s.t.\;\sum_{i\in N}x_{i}\left(t\right)\leq R_{\mathrm{total}},\;x_{i}\left(t\right)\geq 0,$$

where $p_{i}$ is the priority weight. This problem yields a closed-form solution via Lagrange multipliers, in which the optimal allocation $x_{i}^{*}\left(t\right)$ is proportional to the reported demand:

(4) $$x_{i}^{*}\left(t\right)=\frac{d_{i}^{\mathrm{rep}}\left(t\right)\cdot p_{i}}{\sum_{j\in N}d_{j}^{\mathrm{rep}}\left(t\right)\cdot p_{j}}\cdot R_{\mathrm{total}}.$$

**Figure 1:**
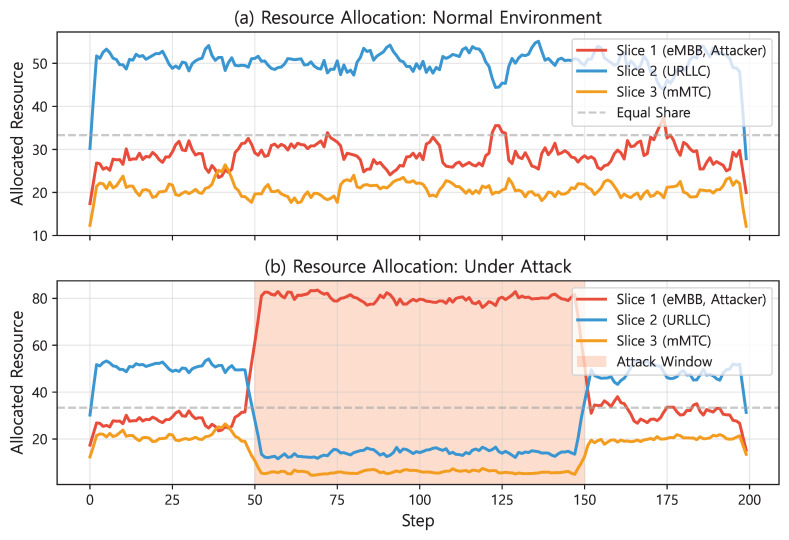
Dynamic resource allocation per slice under normal and attack conditions.

As illustrated in Figure 1, the PF allocator distributes resources equitably under normal conditions, while inflated reports from a single slice can result in monopolistic behavior.

Each slice *i* has a minimum Service Level Agreement (SLA) threshold ${\mathrm{SLA}}_{i}$. The system-wide SLA violation rate, which serves as the primary evaluation metric, is defined as

(5) $$V_{\mathrm{sys}}=\frac{1}{T\cdot |N|}\sum_{t=0}^{T-1}\sum_{i\in N}1\left[x_{i}^{*}\left(t\right)<{\mathrm{SLA}}_{i}\right].$$

The attack-period average of $V_{\mathrm{sys}}$ over $t\in [T_{s},T_{e})$ serves as the basis for comparison among defense methods in Section 4.

### 2.2. Threat Model and Attack Scenarios

This research addresses a resource exhaustion attack that arises from the demand-proportional structure of the centralized PF allocator, considered within the broader context of slice-level security threats [18]. A white-box adversary is assumed to compromise an arbitrary slice a∈N and manipulate its reported demand $d_{a}^{\mathrm{rep}}\left(t\right)$. The adversary’s objective is to maximize

(6) $$\max_{d_{a}^{\mathrm{rep}}\left(t\right)}E\left[x_{a}\left(t\right)+\lambda \sum_{i\in N\setminus \{a\}}1\left[x_{i}\left(t\right)<{\mathrm{SLA}}_{i}\right]\right].$$

The adversary thus seeks to maximize its own physical allocation $x_{a}\left(t\right)$ while inducing resource starvation in the remaining legitimate slices, governed by a penalty weight λ>0.

**Figure 2:**
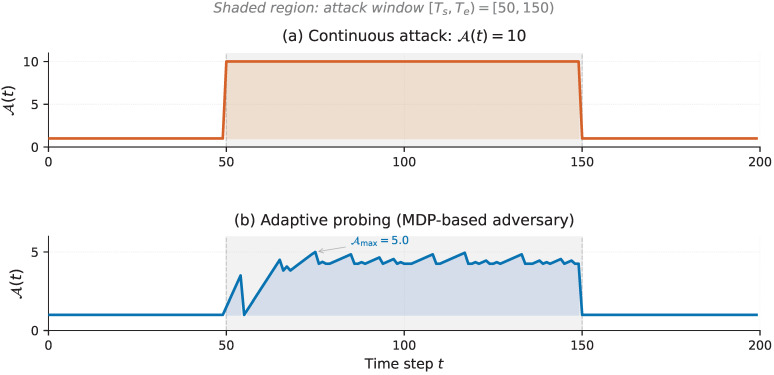
Temporal amplification patterns of the Continuous and Adaptive Probing attack scenarios.

To achieve this adversarial objective, the attacker introduces a dynamic amplification function A(t) such that $d_{a}^{\mathrm{rep}}\left(t\right)=d_{a}^{\mathrm{true}}\left(t\right)\cdot A\left(t\right)$. Two representative scenarios are considered, spanning the spectrum of adversarial sophistication from blunt high-volume threats to MDP-based dynamic evasion [26,27]. For both scenarios, the active attack window is fixed as $\left[T_{s},T_{e}\right)=\left[50,150\right)$, partitioning each episode into warm-up, attack, and post-attack recovery phases. Figure 2 visualizes the temporal patterns of both scenarios.

**Scenario 1: Continuous Attack.** The adversary injects high-intensity demand consistently over the attack window, forming a rectangular pulse pattern (panel a of Figure 2):

(7) $$A\left(t\right)=10.0,\;\forall t\in \left[T_{s},T_{e}\right).$$

Despite its simplicity, this scenario causes catastrophic SLA violations in undefended systems and serves as a foundational stress test for static outlier detection methods.

**Scenario 2: Adaptive Probing Attack.** The adversary approximates a Markov Decision Process (MDP) by observing the allocation feedback $x_{a}\left(t-1\right)$ and dynamically updating A(t) based on whether the previous attack was detected. The detection indicator is defined as

(8) $$I_{D}\left(t\right)=1\left[x_{a}\left(t-1\right)<\rho \cdot d_{a}^{\mathrm{true}}\left(t-1\right)\right],$$

where ρ=0.7 represents the detection tolerance. This value models a moderately cautious adversary that interprets allocations below 70% of the true demand as evidence of defensive intervention. The threshold is chosen to tolerate normal allocation fluctuations caused by coexisting slice bursts, while still allowing the adversary to detect partial mitigation. Values closer to 1.0 would cause spurious backoffs due to natural variability, whereas values closer to 0.5 would make the adversary insensitive to most defensive interventions. Initialized at $A(T_{s})=1.5$, $\Delta (T_{s})=0.5$, and $A_{\mathrm{safe}}\left(T_{s}\right)=1.0$, the attack policy operates as follows: if detected ($I_{D}\left(t\right)=1$), the adversary backs off to the safe state $A\left(t\right)=A_{\mathrm{safe}}\left(t-1\right)$ and reduces the step size $\Delta \left(t\right)=\max(\Delta_{\min},\gamma \cdot \Delta \left(t-1\right))$ with γ=0.7 and $\Delta_{\min}=0.1$; if undetected ($I_{D}\left(t\right)=0$), the adversary increments $A\left(t\right)=\min(A_{\max},A\left(t-1\right)+\Delta \left(t-1\right))$ up to the upper bound $A_{\max}=5.0$. After κ=5 consecutive undetected steps, a new safe baseline $A_{\mathrm{safe}}\left(t\right)=A\left(t-1\right)$ is established. The resulting increment-and-backoff trajectory is visible in panel b of Figure 2.

The final reported demand incorporates additive deception noise $\eta \left(t\right)∼N\left(0,\sigma_{\mathrm{att}}^{2}\right)$ with $\sigma_{\mathrm{att}}=0.5$ to mimic natural traffic variability:

(9) $$d_{a}^{\mathrm{rep}}\left(t\right)=d_{a}^{\mathrm{true}}\left(t\right)\cdot A\left(t\right)+\eta \left(t\right).$$

**Figure 3:**
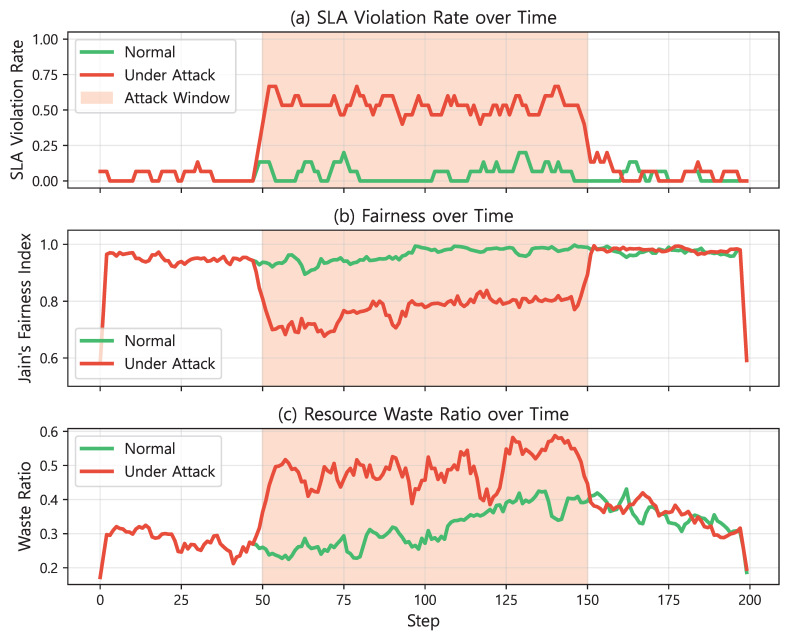
Comparison of system performance metrics under normal and attack conditions.

The system-level impact of such attacks is illustrated in Figure 3. During the attack window, adversarial demand manipulation increases SLA violations, collapses fairness, and raises resource waste, demonstrating a system-wide degradation beyond the compromised slice.

These two scenarios represent the two extreme ends of practical adversarial sophistication: Continuous attacks rely on overwhelming volume, whereas Adaptive Probing attacks rely on stealthy escalation calibrated to defense feedback. Defense mechanisms performing well across both are likely to provide adaptive baseline protection in real 5G/6G deployments. To keep the threat model analytically controlled, this study sets the adversarial slice as a=1, corresponding to the eMBB slice, which provides a high-impact attack case because of its relatively large demand contribution as detailed in Section 4.1.

This single-slice setting allows us to isolate the effect of demand inflation under PF allocation; however, coordinated multi-slice attacks may also occur in practical deployments. In such cases, compromised sub-slices can split the amplification load, reducing per-slice anomaly magnitude and weakening purely spatial outlier detection. The LSTM-AE stage can provide partial visibility by modeling the temporal evolution of the joint demand matrix, but robust collusion detection would require correlation-aware indicators, such as inter-slice demand shifts, aggregate imbalance, and fairness degradation. We therefore identify multi-slice collusion detection as an important extension of the current framework.

## 3. Materials and Methods

### 3.1. Overview and Defense Philosophy

AutoGuard-Hybrid is designed as an unsupervised detection-to-mitigation framework that secures 5G/6G network slicing against resource exhaustion attacks, where a malicious slice inflates its reported demand to obtain a disproportionate share of shared resources. The framework combines Isolation Forest and LSTM-Autoencoder as complementary unsupervised detectors with allocation-aware mitigation policies, so that anomaly scores are directly translated into demand purification actions before resource allocation. The framework is built upon three core principles. First, AutoGuard-Hybrid operates as a non-intrusive functional wrapper F that precedes the baseline allocator and requires no structural modifications to the underlying 5G core network. Second, the cascade detection front-end produces complementary signals across spatial and temporal regimes, while the mitigation policy translates these signals into adaptive resource-control actions whose intensity scales inversely with the detected threat level. Third, the framework ensures resilience against zero-day attacks through unsupervised optimization on normal traffic profiles, eliminating the need for labeled attack data.

**Figure 4:**
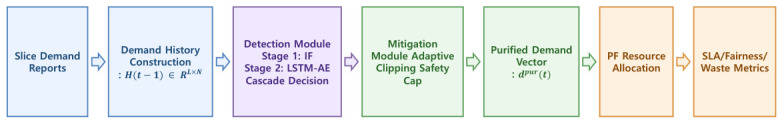
Overall system architecture of the proposed AutoGuard-Hybrid detection-to-mitigation framework. Colored blocks indicate the main functional stages, and arrows represent the data and control flow.

Figure 4 illustrates the overall system architecture of the proposed AutoGuard-Hybrid framework. Slice demand reports are first transformed into a historical demand matrix, which is then processed by the cascade detection module composed of Isolation Forest and LSTM-AE. The resulting anomaly evidence is converted into demand purification actions through Adaptive Clipping and a Safety Cap before being delivered to the PF resource allocator.

### 3.2. System Model and Threat Formulation

Building on the network slicing environment defined in Section 2.1, the multivariate observation vector collected by the resource allocator at time *t* is

(10) $$o\left(t\right)={\left[d_{1}^{\mathrm{obs}}\left(t\right),d_{2}^{\mathrm{obs}}\left(t\right),\ldots ,d_{N}^{\mathrm{obs}}\left(t\right)\right]}^{⊤}\in R^{N}.$$

For attack scenarios in Section 2.2, an adversary controlling slice a∈N manipulates its reported demand piecewise:

(11) $$d_{a}^{\mathrm{rep}}\left(t\right)=\left\{\begin{array}{cc} d_{a}^{\mathrm{true}}\left(t\right)\cdot A\left(t\right)+\eta \left(t\right), & \mathrm{if}\;t\in [T_{s},T_{e})\\ d_{a}^{\mathrm{true}}\left(t\right), & \mathrm{otherwise}, \end{array}\right.$$

where A(t)≥1 is the dynamic amplification factor and $\eta \left(t\right)∼N\left(0,\sigma_{\mathrm{att}}^{2}\right)$ is the deception noise.

The objective of the defense framework F is to map the manipulated observations into a purified vector $o^{\prime }\left(t\right)$ using both the current observation and the temporal history matrix:

(12) $$o^{\prime }\left(t\right)=F\left(o\left(t\right),H\left(t-1\right)\right),\;H\left(t-1\right)={\left[o\left(t-L\right),\ldots ,o\left(t-1\right)\right]}^{⊤}\in R^{L\times N},$$

with lookback window L=10. Following purification, the PF allocator computes the final allocation securely as defined in Section 2.1, with $d_{i}^{\mathrm{rep}}$ replaced by $o_{i}^{\prime }$.

### 3.3. Cascade Detection Architecture

The cascade detection architecture serves as the front-end of the AutoGuard-Hybrid framework, providing complementary anomaly signals that drive the subsequent mitigation policy. Rather than acting as a standalone classification module, the cascade integrates two unsupervised detectors with different inductive biases: Isolation Forest for static spatial anomalies and LSTM-Autoencoder for temporal pattern deviations. The detection outputs of both stages are passed to the mitigation policy described in Section 3.4, where they are translated into resource-control actions.

#### 3.3.1. Stage 1: Static Spatial Isolation

The first layer employs the Isolation Forest (IF) algorithm with an ensemble of M=100 binary space-partitioning trees to identify extreme outliers that deviate significantly from the multidimensional expectation of normal traffic. The threat score is derived from the average isolation path length:

(13) $$s_{\mathrm{IF}}\left(o\left(t\right)\right)=2^{-\frac{E[h(o(t))]}{c(\psi )}},$$

where E[h(o(t))] is the average path length across the ensemble (a shorter length indicates a more anomalous position), and c(ψ)=2H(ψ−1)−2(ψ−1)/ψ is the normalization factor with H(·) denoting the harmonic number. The detection flag is activated when the score exceeds a threshold $\tau_{\mathrm{IF}}$ derived from contamination parameter $\nu_{\mathrm{IF}}=0.01$:

(14) $$y_{\mathrm{IF}}\left(t\right)=1\left[s_{\mathrm{IF}}\left(o\left(t\right)\right)>\tau_{\mathrm{IF}}\right].$$

#### 3.3.2. Stage 2: Temporal Deception Detection

Static analysis alone is insufficient against adversaries that dynamically calibrate intensity to remain within normal spatial boundaries. The second layer therefore employs an LSTM-Autoencoder to verify non-linear temporal dependencies. Given the input tensor $X\left(t\right)\in R^{L\times N}$, the model reconstructs the sequence as

(15) $$\hat{X}\left(t\right)=f_{\mathrm{dec}}\left(f_{\mathrm{enc}}\left(X\left(t\right);\theta_{\mathrm{enc}}\right);\theta_{\mathrm{dec}}\right).$$

The encoder is composed of a single LSTM layer with hidden size h=16, followed by a fully connected layer projecting to latent dimension $d_{z}=4$. The decoder reverses this architecture: a fully connected layer expands the latent representation back to dimension *h*, replicates it across *L* time steps, and processes through an LSTM layer producing the reconstructed output of dimension *N*. The complete architecture has 1744 parameters, enabling efficient real-time inference. Although the per-step observation vector o(t) has only N=3 dimensions, the effective input to the LSTM-AE is the spatiotemporal tensor $X\left(t\right)\in R^{L\times N}$, whose dimension is L×N=30 with L=10. Therefore, the role of the LSTM-AE is not high-dimensional feature extraction from a single observation, but temporal sequence modeling over the recent demand history. The temporal axis carries discriminative information for distinguishing gradual amplification patterns from legitimate burst variations. The compact architecture described above reduces the risk of overfitting on low-dimensional traffic data, and the threshold calibration on a held-out validation set further constrains the model behavior to the variability range of normal traffic. The model is trained by minimizing the mean squared reconstruction error:

(16) $$L\left(\theta_{\mathrm{enc}},\theta_{\mathrm{dec}}\right)=\frac{1}{|D_{\mathrm{train}}|}\sum_{X\in D_{\mathrm{train}}}{∥X-f_{\mathrm{dec}}\left(f_{\mathrm{enc}}\left(X\right)\right)∥}_{F}^{2},$$

where ${∥\cdot ∥}_{F}$ denotes the Frobenius norm. The temporal threat score is the per-element MSE between original and reconstructed sequences:

(17) $$s_{\mathrm{LSTM}}\left(t\right)=\frac{1}{L\cdot N}\sum_{l=1}^{L}\sum_{i=1}^{N}{\left(X_{l,i}\left(t\right)-{\hat{X}}_{l,i}\left(t\right)\right)}^{2}.$$

The detection threshold is fixed at the 99th percentile of reconstruction errors on a held-out validation set, $\tau_{\mathrm{LSTM}}=Q_{0.99}\left(\left\{s_{\mathrm{LSTM}}\left(X\right):X\in D_{\mathrm{val}}\right\}\right)$, ensuring an approximate 1% false positive rate. The detection flag is activated as $y_{\mathrm{LSTM}}\left(t\right)=1\left[s_{\mathrm{LSTM}}\left(t\right)>\tau_{\mathrm{LSTM}}\right]$.

#### 3.3.3. Rationale for Regime-Aware Cascade Design

The cascade design exploits the complementary detection regimes of static and temporal methods. Let $A_{\mathrm{spatial}}$ denote attacks producing values in the tail of the marginal distribution $P(o_{i})$, and $A_{\mathrm{temporal}}$ denote attacks that preserve marginal distributions but alter joint distributions P(o(t−L+1),…,o(t)). Isolation Forest is particularly effective for $A_{\mathrm{spatial}}$ but offers limited sensitivity to temporal dependencies; conversely, LSTM-Autoencoder captures sequential patterns in $A_{\mathrm{temporal}}$ but provides weaker spatial discrimination. Real-world attacks satisfy $A_{\mathrm{real}}\subseteq A_{\mathrm{spatial}}\cup A_{\mathrm{temporal}}$, so a single detector can address only one regime, while the cascade design covers their union. This is well-aligned with the two attack scenarios considered in this work: Continuous attacks fall into $A_{\mathrm{spatial}}$ and are detected by IF, while Adaptive Probing attacks span both regimes through temporal evasion patterns combined with periodic spatial spikes. This complementary coverage provides the design rationale for the regime-dependent performance demonstrated in Section 4.

### 3.4. Adaptive Threat Mitigation

The core mechanism of AutoGuard-Hybrid is adaptive threat mitigation rather than simple binary detection. Defense intensity is modulated piecewise based on the joint state of $y_{\mathrm{IF}}$ and $y_{\mathrm{LSTM}}$. This logic is supported by two complementary mechanisms: a Safety Cap that prevents extreme resource monopolization and an Adaptive Clipping Ratio that adjusts mitigation intensity according to the detected threat level.

An absolute upper bound is established from empirical normal-traffic statistics to prevent any slice from monopolizing resources:

(18) $$c_{i}=\left(\mu_{i}+3\sigma_{i}\right)\cdot \beta ,$$

where β=1.2 is a margin factor that accommodates legitimate burst traffic. The use of $\mu_{i}+3\sigma_{i}$ should be interpreted as a lightweight empirical scale bound rather than a strict Gaussian confidence interval. Although the $3\sigma_{i}$ term has a conventional Gaussian interpretation, the additional margin factor β=1.2 provides tolerance for legitimate burst variability by setting the actual cap to $c_{i}=\left(\mu_{i}+3\sigma_{i}\right)\cdot \beta$. Thus, moderate bursts above $\mu_{i}+3\sigma_{i}$ can still be accommodated, while only extreme reports exceeding $c_{i}$ are demoted to the normal mean $\mu_{i}$ when mitigation is triggered. Here, β should not be directly compared with the burst multiplier $M_{i}^{\mathrm{burst}}$ because it scales the empirical bound $\mu_{i}+3\sigma_{i}$ rather than the mean demand $\mu_{i}$. Thus, legitimate burst traffic can remain below the Safety Cap even when $M_{i}^{\mathrm{burst}}>\beta$, and cap-based demotion is applied only when an excessive report is also detected as abnormal.

In the experimental configuration, the Bernoulli-triggered burst model defined in Section 2.1, with burst probabilities up to $P_{\mathrm{burst}}=0.1$ and burst multipliers up to $M_{\mathrm{burst}}=1.8$, produces burst variations that are empirically absorbed by the β-scaled cap. For environments dominated by non-Gaussian long-tail IoT traffic, the Safety Cap module can be replaced by robust data-driven bounds, such as a percentile-based cap or a median absolute deviation (MAD)-based cap, without modifying the overall detection-to-mitigation pipeline. In this work, the mean-variance form is adopted for computational simplicity and compatibility with the 1 ms TTI constraint, while the modularity of the framework preserves its applicability to heavier-tailed traffic distributions through alternative cap formulations.

For traffic identified as anomalous but below the Safety Cap, a stage-specific clipping ratio is applied:

(19) $$r_{i}\left(t\right)=\max\left(r_{\min},\min\left(1,\frac{\tau_{*}}{s_{*}\left(t\right)+\epsilon }\right)\right),$$

where $\tau_{*}$ and $s_{*}$ correspond to the triggering stage, either IF or LSTM, and $\epsilon =10^{-8}$ prevents division by zero. The minimum clipping ratio $r_{\min}$ is set to 0.05 for both stages, ensuring that anomaly scores significantly above the threshold result in clipping the reported demand to a small fraction of its original value. This unified setting maintains the inverse-proportional scaling between the detected threat level and the applied clipping, while preventing detected attackers from sustaining meaningful resource allocation through a high lower bound.

The purified observation $o^{\prime }\left(t\right)$ is then determined by one of three branches. In Strong Control, when $y_{\mathrm{IF}}=1$, demands above $c_{i}$ are demoted to $\mu_{i}$, while the remaining demands are clipped through the inverse-proportional ratio with $r_{\min}=0.05$. In Adaptive Control, when $y_{\mathrm{IF}}=0$ and $y_{\mathrm{LSTM}}=1$, the same inverse-proportional clipping is applied with $r_{\min}=0.05$, where the per-slice LSTM-AE score determines the clipping intensity on a per-slice basis. In Transparent Pass, when both flags are zero, traffic is forwarded unchanged so that $o_{i}^{\prime }\left(t\right)=o_{i}\left(t\right)$.

### 3.5. Algorithm Description and Training Procedure

**Figure 5:**
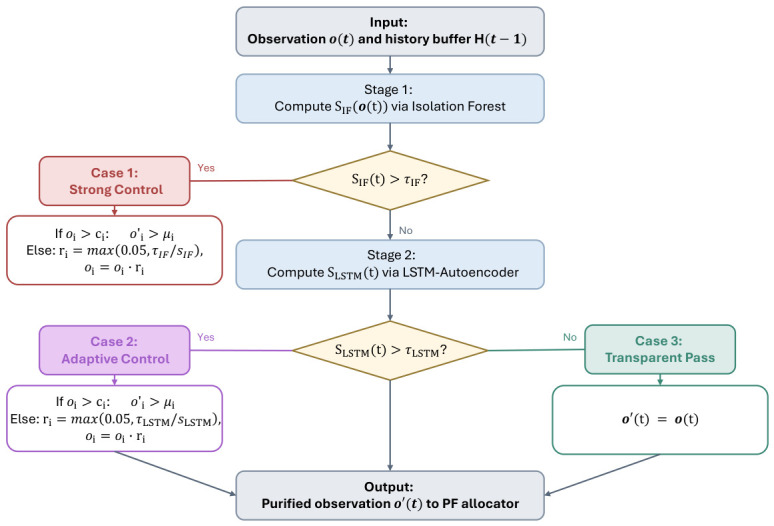
Cascade decision logic of AutoGuard-Hybrid, where observations are sequentially processed by IF and LSTM-AE to apply spatial mitigation, temporal mitigation, or transparent pass-through before PF allocation. Colored blocks denote decision stages, and arrows indicate the processing flow.

The complete operational logic of AutoGuard-Hybrid is illustrated in Figure 5, which visualizes the cascade decision pathway from input observation to purified output through three sequential decision points.

The framework is trained offline in an unsupervised manner using only normal observation data $D_{\mathrm{normal}}$. Empirical statistics $\left(\mu_{i},\sigma_{i}\right)$ and Safety Cap bounds $c_{i}$ are computed directly from $D_{\mathrm{normal}}$. The Stage 1 IF detector is fitted with M=100 trees and contamination $\nu_{\mathrm{IF}}=0.01$. The Stage 2 LSTM-AE detector is trained for E=50 epochs using the Adam optimizer with learning rate $\alpha =10^{-3}$ and batch size 64 on length-*L* sequences extracted from $D_{\mathrm{normal}}$, partitioned into 80% training and 20% validation sets. The LSTM threshold is calibrated as $\tau_{\mathrm{LSTM}}=Q_{0.99}\left(\left\{s_{\mathrm{LSTM}}\left(X\right):X\in D_{\mathrm{val}}\right\}\right)$, ensuring approximately 1% false positive rate.

### 3.6. Computational Complexity and Operational Feasibility

For deployment in practical 5G/6G environments, the defense wrapper must satisfy the strict 1 ms Transmission Time Interval (TTI) constraint. The IF spatial isolation search is bounded by O(MlogK) for *K* training samples and *M* trees, while the LSTM-AE forward pass complexity is approximately $O(L\left(h^{2}+Nh\right))$, where the Nh term accounts for input and output projections associated with the slice dimension. The asymptotic per-step time complexity is therefore

(20) $$O(M\log K+L\left(h^{2}+Nh\right)).$$

With the experimental configuration (M=100, K=10,000, L=10, N=3, h=16), measured inference time on standard CPU hardware remains well under 1 ms per step, demonstrating real-time feasibility within the considered 5G configuration.

Although the experiments use three representative slices corresponding to eMBB, URLLC, and mMTC, the proposed framework is not structurally restricted to N=3. The history matrix $H\left(t-1\right)\in R^{L\times N}$ increases linearly with the number of slices when the lookback length *L* is fixed. Similarly, the LSTM-AE input and output dimensions scale with *N*, while the recurrent hidden dimension *h* can remain compact. Therefore, with fixed *L* and *h*, the additional computational cost of the temporal stage grows approximately linearly with the number of slices. The reported 1744-parameter LSTM-AE corresponds to the three-slice configuration used in this study; in denser slicing deployments, the model can be re-instantiated with the corresponding input dimension while preserving the same lightweight design principle. We therefore view dense multi-slice scaling as an implementation-dependent extension, where the sub-1 ms inference constraint should be verified under the target gNB hardware and slice density.

## 4. Simulation Results and Discussion

### 4.1. Simulation Setup

The 5G/6G network slicing environment defined in Section 2 is simulated with $R_{\mathrm{total}}=100$ and T=200 time steps per episode. To improve statistical robustness, each evaluation is repeated over 20 independent environment seeds, ranging from 42 to 61. These seeds affect stochastic traffic generation, burst occurrence, measurement noise, deception noise, and attack-trajectory realizations. Unless otherwise stated, scalar performance metrics are reported as the mean and standard deviation across the 20 repeated runs, with 95% confidence intervals additionally used for error bars and confidence bands in the figures.

The detection models are trained purely on an offline dataset $D_{\mathrm{normal}}$ containing K=10,000 normal observations generated under the slice profiles in Table 2, with no reliance on labeled attack data. The trained defenders are fixed and shared across all evaluation seeds, reflecting a deployment setting in which the defense model is trained offline and then evaluated under multiple stochastic traffic and attack realizations. The dataset is partitioned into 80% training and 20% validation for the LSTM-Autoencoder, while the Isolation Forest uses the entire dataset. All experiments are conducted in Python 3.12 with PyTorch 2.0 and scikit-learn 1.3 on a standard CPU environment.

**Figure 6:**
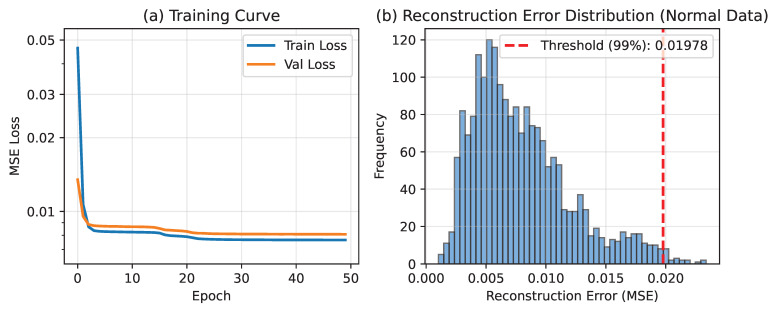
Training and validation loss curves of the LSTM-Autoencoder.

The Stage 1 Isolation Forest is fitted in a single pass using random space partitioning and therefore does not produce a learning curve, so Figure 6 reports only the convergence of the Stage 2 LSTM-Autoencoder.

The adversarial slice is set as a=1, corresponding to the eMBB slice, which has the highest mean demand of $\mu_{1}=30$ and the largest variance among the three slices. This configuration provides a high-impact attack case because manipulated eMBB demand can strongly distort PF allocation. In this evaluation, the mMTC slice represents large-scale IoT sensor traffic, so reductions in $V_{\mathrm{sys}}$ indicate improved protection of slice-level availability for coexisting sensing and monitoring services.

Three primary metrics are employed. The system-wide SLA violation rate $V_{\mathrm{sys}}$, defined in Section 2.1, serves as the primary metric. Two complementary metrics measure resource distribution quality: Jain’s Fairness Index J∈[1/|N|,1], where 1 indicates perfect fairness, and the Resource Waste Ratio W. The two metrics are defined as

(21) $$J\left(t\right)=\frac{{\left(\sum_{i\in N}x_{i}\left(t\right)/d_{i}^{\mathrm{true}}\left(t\right)\right)}^{2}}{\left|N\right|\cdot \sum_{i\in N}{\left(x_{i}\left(t\right)/d_{i}^{\mathrm{true}}\left(t\right)\right)}^{2}},\;W\left(t\right)=\frac{\sum_{i\in N}\max\left(0,x_{i}\left(t\right)-d_{i}^{\mathrm{true}}\left(t\right)\right)}{R_{\mathrm{total}}}.$$

AutoGuard-Hybrid is benchmarked against five configurations spanning the spectrum of unsupervised defense techniques [12,28]: No Defense, in which raw observations are passed directly to the allocator; Z-Score thresholding with $|z_{i}|>3$ [12]; Isolation Forest with M=100 trees and ν=0.01 [19]; One-Class SVM with an RBF kernel and ν=0.01 [20]; and AutoGuard, the LSTM-only ablation of the proposed framework [21,28]. To ensure a fair comparison, all baselines incorporate the same Adaptive Clipping and Safety Cap mechanisms described in Section 3.4, differing only in their detection components.

### 4.2. Ablation Study on Mitigation Mechanisms

To clearly disentangle the effect of each mitigation mechanism, namely Adaptive Clipping and Safety Cap, from detector heterogeneity, the ablation is conducted using the LSTM-only AutoGuard variant under the Continuous Attack scenario. The contribution of the cascade architecture itself is separately evaluated in Section 4.3.

As shown in Table 3, the ablation reveals two key insights. First, the equivalence of A0 and A1 shows that anomaly detection alone is insufficient to improve resource allocation, because the system requires an explicit mitigation mapping to alter suspicious demand reports before PF scheduling. Second, the transition from A1 to A2 reduces $V_{\mathrm{sys}}$ from 0.5368 to 0.1342, corresponding to a 75.0% reduction achieved by score-proportional Adaptive Clipping alone. The full LSTM-based mitigation pipeline further reduces $V_{\mathrm{sys}}$ to 0.0827, achieving an overall 84.6% reduction relative to the no-defense baseline. These findings confirm that the mitigation framework, namely Adaptive Clipping combined with the Safety Cap, is the main source of system-level resilience. The complementary detection benefit of adding Stage 1 IF on top of this mitigation foundation is quantified separately in Section 4.3.

### 4.3. Comprehensive Performance Comparison

**Figure 7:**
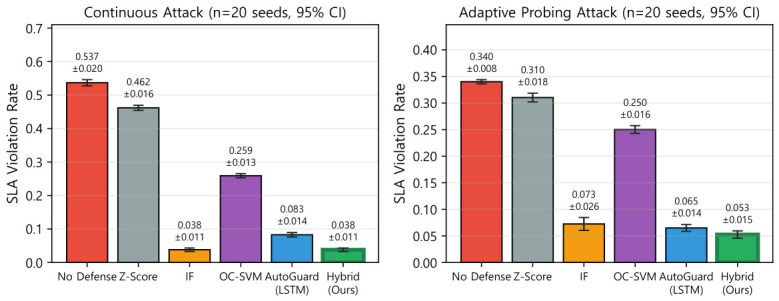
System-wide SLA violation rates under Continuous and Adaptive Probing attacks. Bars indicate mean values over 20 random seeds, and error bars denote 95% confidence intervals.

AutoGuard-Hybrid is evaluated against the five baselines across both attack scenarios formulated in Section 2.2. The evaluation considers three system-level metrics: the system-wide SLA violation rate $V_{\mathrm{sys}}$, Jain’s Fairness Index J, and Resource Waste Ratio W. These metrics capture different aspects of network operation, where $V_{\mathrm{sys}}$ measures SLA preservation, J quantifies the equity of resource distribution across slices, and W measures the efficiency of resource utilization. Figure 7 compares the system-wide SLA violation rates of the defense methods under Continuous and Adaptive Probing attacks.

The results in Table 4 and Table 5 reveal a notable distinction between the two attack scenarios. Under the Continuous Attack, the high-volume rectangular pulse pattern produces clear spatial outliers that are readily isolated by the Stage 1 IF detector alone. Consequently, AutoGuard-Hybrid and Isolation Forest achieve the same mean $V_{\mathrm{sys}}$ of 0.0380, with the Stage 2 LSTM-AE providing only marginal supplementary detections in this regime. This indicates that for blunt high-magnitude attacks, a single static spatial detector is sufficient, and the cascade’s contribution is to confirm rather than extend the detection coverage. This result also suggests a practical optimization opportunity. Under extremely high-intensity blind attacks, where the Stage 1 IF detector produces a confident detection trigger, the LSTM-AE forward pass could be skipped through short-circuit evaluation, allowing the framework to directly proceed to the Strong Control branch. Such an implementation would reduce computational overhead in obvious spatial-outlier regimes without changing the mitigation behavior. However, this shortcut should be applied only when the Stage 1 confidence is sufficiently high, because the LSTM-AE stage remains necessary for Adaptive Probing attacks, where temporal deviations are more subtle and may not be captured by static spatial detection alone.

The Adaptive Probing scenario, however, demonstrates the benefit of coupling temporal anomaly evidence with the mitigation policy. The MDP-based adversary deliberately calibrates its amplification factor to remain within normal spatial boundaries between escalation phases, which weakens the discriminative power of static methods. Isolation Forest alone achieves a mean $V_{\mathrm{sys}}$ of 0.0725, whereas AutoGuard-Hybrid reduces this to 0.0525, corresponding to a 27.6% improvement in the mean system-wide SLA violation rate. This gain originates from the Stage 2 LSTM-AE, which captures temporal escalation patterns that IF cannot detect from static observations alone. AutoGuard-Hybrid also achieves the highest mean J of 0.9691 in this scenario, reflecting more equitable resource distribution.

These observations together support the central design principle of AutoGuard-Hybrid: a single-method defense is sufficient only when the attack pattern aligns with that method’s detection regime, while sophisticated adversaries that deliberately span multiple regimes require complementary detection mechanisms. The cascade’s value therefore lies in providing robust protection across the full spectrum of adversarial sophistication, from blunt volume attacks where static spatial detection suffices, to subtle temporal manipulation where temporal modeling becomes indispensable.

The trade-off between $V_{\mathrm{sys}}$ and W observed in the LSTM-only variant under the Continuous Attack reflects an additional aspect of this design. AutoGuard with LSTM only achieves a lower mean W of 0.2569 compared to AutoGuard-Hybrid at 0.3093, but this comes at the cost of a higher mean $V_{\mathrm{sys}}$ of 0.0827 versus 0.0380. This is consistent with the safety-first design philosophy of 5G/6G slicing, where SLA preservation for safety-critical slices such as URLLC for autonomous driving and remote surgery takes precedence over marginal efficiency losses.

**Figure 8:**
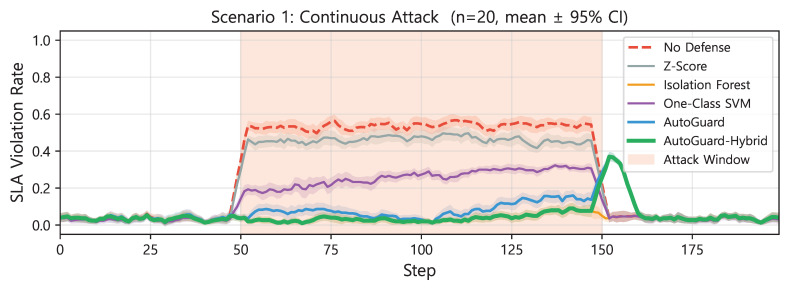
Mean temporal evolution of $V_{\mathrm{sys}}$ under the Continuous Attack scenario over 20 random seeds. Shaded bands indicate 95% confidence intervals.

**Figure 9:**
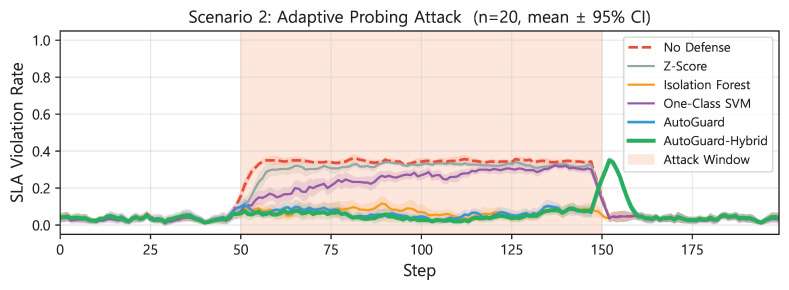
Mean temporal evolution of $V_{\mathrm{sys}}$ under the Adaptive Probing Attack scenario over 20 random seeds. Shaded bands indicate 95% confidence intervals.

The temporal dynamics of $V_{\mathrm{sys}}$ are illustrated in Figure 8 and Figure 9. The contrast between the two scenarios is clearly visible: under the Continuous Attack, the IF and Hybrid trajectories overlap closely throughout the attack window, whereas under Adaptive Probing, AutoGuard-Hybrid maintains a lower mean violation rate than IF, particularly during the gradual escalation phases where the temporal stage actively contributes.

### 4.4. Stage Activation Analysis and Cascade Synergy

To examine the complementary synergy of the cascade stages, the activation distribution across the 200-step episodes is analyzed in Table 6. Each step is classified into Stage 1 IF detected, Stage 2 LSTM-only detected, Normal where both stages are clear, or Warm-up. The reported values indicate mean activation counts over 20 random seeds.

The activation statistics quantitatively confirm the regime-dependent behavior identified in Section 4.3. Under the Continuous Attack, Stage 1 IF dominates with an average of 101.0 activations, while Stage 2 LSTM activates only 9.3 times on average. Thus, approximately 91.6% of all detections originate from the static spatial stage. Because the rectangular pulse pattern of the Continuous Attack produces unambiguous spatial outliers, IF detects nearly every attack step on its own.

Under the Adaptive Probing Attack, the activation pattern shifts substantially. Stage 1 IF activations decrease to 72.9 on average, while Stage 2 LSTM activations increase to 29.4, accounting for approximately 28.7% of all detections. This shift reflects the adversary’s deliberate strategy of remaining within normal spatial boundaries between escalation phases, which transfers a significant portion of the detection burden to the temporal stage. Without Stage 2, the attack steps captured by LSTM-AE would propagate through the allocator unfiltered, leading to the higher $V_{\mathrm{sys}}$ observed for the IF baseline in Table 5.

This activation pattern shows that the cascade design adapts naturally to the attack regime: the static stage suffices for blunt threats, while the temporal stage becomes more important against sophisticated adversaries. The overall result is robust protection across the spectrum of attack sophistication, achieved without any explicit knowledge of the attack type at inference time.

### 4.5. Practicality and Real-Time Feasibility

The wrapper-based architecture described in Section 3.1 enables integration into existing 5G core infrastructures with no modification to the underlying allocator. As established by the complexity analysis in Section 3.6, the per-step inference time remains well below the 1 ms TTI constraint, which confirms the framework’s suitability for real-time deployment.

Beyond computational feasibility, the unsupervised formulation provides additional deployment advantages. Because all detection thresholds and mitigation parameters are derived solely from normal traffic statistics, the framework adapts to heterogeneous slice profiles without requiring labeled attack data or periodic retraining. This property is particularly advantageous for 6G deployments, where new service types and attack vectors are expected to emerge continuously, rendering supervised approaches difficult to maintain. The cascade structure also allows the two detection stages to be updated or replaced independently as new anomaly detection methods become available, supporting long-term extensibility of the framework. This deployability is particularly relevant to 5G/6G-enabled IoT sensor networks, where lightweight and real-time resource protection is required to maintain reliable sensing-data delivery under evolving attack patterns.

### 4.6. Robustness Discussion Under Legitimate Traffic Surges

Beyond the attack scenarios evaluated above, a practical deployment concern is whether legitimate large-scale traffic surges may be misinterpreted as adversarial demand inflation. In highly dynamic 5G/6G environments, such surges may occur due to emergency IoT broadcasts, stadium-scale sensing events, or sudden monitoring bursts. These flash-crowd events can temporarily increase reported demand and temporal reconstruction errors, making them appear similar to anomaly patterns observed during resource exhaustion attacks. However, unlike adversarial demand inflation, these surges originate from actual service requests rather than strategic manipulation.

AutoGuard-Hybrid mitigates this risk in two ways. First, the mitigation policy is not triggered solely by a single temporal deviation but is applied through the cascade decision logic together with the Safety Cap and Adaptive Clipping. Legitimate bursts that remain within the empirical Safety Cap are tolerated, while extreme reports exceeding the cap are controlled only when the detector indicates abnormality. Second, the Adaptive Clipping ratio is score-proportional, so marginal threshold crossings induce weaker demand reduction than high-confidence anomalies. This prevents sudden but moderate real traffic increases from being treated in the same way as persistent adversarial demand inflation.

Nevertheless, distinguishing flash crowds from intelligent attacks remains a practical deployment challenge. In operational 5G/6G systems, this ambiguity can be reduced by incorporating auxiliary context signals, such as event-triggered service notifications, cross-slice burst correlation, admission-control indicators, and short-term persistence checks. These signals can be used as additional confidence filters before applying strong mitigation so that correlated legitimate demand surges are less likely to be treated as single-slice or strategically timed demand manipulation.

## 5. Conclusions

This paper proposed AutoGuard-Hybrid, an unsupervised detection-to-mitigation framework for mitigating resource exhaustion attacks in 5G/6G network slicing environments that support heterogeneous services, including mMTC-based IoT sensor slices. The framework couples complementary unsupervised detectors with allocation-aware mitigation policies, translating anomaly scores into demand purification actions through Adaptive Clipping and a Safety Cap before resource allocation. By preventing manipulated demand reports from monopolizing shared resources, AutoGuard-Hybrid aims to preserve slice-level SLA integrity and service availability for coexisting sensing, monitoring, and mission-critical applications.

Experimental results across Continuous and Adaptive Probing attacks show that AutoGuard-Hybrid achieves mean $V_{\mathrm{sys}}$ values of 0.0380 and 0.0525, respectively. The proposed framework remains comparable to Isolation Forest under Continuous attacks, where clear spatial outliers can be handled by static detection alone, while achieving a 27.6% improvement in the mean system-wide SLA violation rate under sophisticated Adaptive Probing attacks. In the Adaptive Probing scenario, Stage 2 LSTM-AE activations increase from 9.3 to 29.4 on average compared with the Continuous scenario. This regime-dependent pattern shows that the cascade front-end adapts naturally to the attack regime, providing complementary detection signals across different attack patterns.

The ablation study further shows that score-proportional Adaptive Clipping alone reduces the system-wide SLA violation rate by 75.0%, while the full mitigation pipeline achieves an 84.6% total reduction. This confirms that the mitigation policy, rather than the detection front-end alone, serves as the main source of system-level resilience. This finding indicates that securing next-generation network slicing requires a transition from binary anomaly classification toward continuous and adaptive threat mitigation. With computational complexity within the 1 ms TTI constraint, AutoGuard-Hybrid offers a practical unsupervised detection-to-mitigation framework for 5G and emerging 6G slicing infrastructures that require reliable resource availability for IoT sensor networks and other latency- or reliability-sensitive services.

Future work will extend AutoGuard-Hybrid toward more expressive temporal and inter-slice modeling. Attention-based sequence models, such as Transformer-style architectures, can be investigated to capture long-range temporal dependencies in slice demand evolution more effectively than recurrent models. In addition, generative models may be used to learn richer normal-demand distributions and synthesize diverse benign traffic patterns for more robust anomaly threshold calibration. These generative traffic models can also support context-aware flash-crowd discrimination by helping distinguish legitimate massive traffic surges from strategically manipulated demand inflation. Another promising direction is graph-based inter-slice modeling, which can capture correlation shifts among coexisting slices and support the detection of coordinated multi-slice resource exhaustion attacks. These extensions would further strengthen the adaptability of detection-to-mitigation frameworks for dynamic and large-scale 5G/6G network slicing environments.

## Figures and Tables

**Table 1:** Summary of key notation used in this paper.

Notation	Description
*N*	Number of network slices
N	Set of slices
*i*	Slice index
*t*	Discrete time step
$R_{\mathrm{total}}$	Total normalized resource capacity
$d_{i}^{\mathrm{true}}\left(t\right)$	True traffic demand of slice *i*
$d_{i}^{\mathrm{rep}}\left(t\right)$	Reported demand of slice *i*
$d_{i}^{\mathrm{obs}}\left(t\right)$	Observed demand with measurement noise
$p_{i}$	Priority weight of slice *i*
$x_{i}\left(t\right)$	Resource allocated to slice *i*
${\mathrm{SLA}}_{i}$	Minimum SLA threshold of slice *i*
$V_{\mathrm{sys}}$	System-wide SLA violation rate
J	Jain’s fairness index
W	Resource waste ratio
A(t)	Attack amplification factor
ρ	Detection tolerance of the adaptive adversary
H(t−1)	Historical demand matrix
*L*	Lookback window length
$s_{\mathrm{IF}}\left(t\right)$	Isolation Forest anomaly score
$s_{\mathrm{LSTM}}\left(t\right)$	LSTM-AE reconstruction anomaly score
$\tau_{\mathrm{IF}}$	Detection threshold of Isolation Forest
$\tau_{\mathrm{LSTM}}$	Detection threshold of LSTM-AE
$c_{i}$	Safety Cap for slice *i*
β	Safety Cap margin factor
$r_{i}\left(t\right)$	Adaptive clipping ratio

**Table 2:** Service profile parameters for the three network slices.

ID	Type	$\mu_{i}$	$\sigma_{i}$	${\mathrm{SLA}}_{i}$	$p_{i}$	Burst $\left(P_{i}^{\mathrm{burst}},M_{i}^{\mathrm{burst}}\right)$
1	eMBB	30.0	5.0	18.0	1.0	(0.10, 1.8)
2	URLLC	22.0	2.0	15.0	2.5	(0.02, 1.3)
3	mMTC	28.0	4.0	17.0	0.8	(0.05, 1.4)

**Table 3:** Ablation study on mitigation components using the LSTM-AE baseline under the Continuous Attack scenario. Values are reported as mean (±standard deviation) over 20 random seeds.

Configuration	Detection	Adaptive Clipping	Safety Cap	$V_{\mathrm{sys}}$
A0: Baseline, No Defense	×	×	×	0.5368(±0.0197)
A1: Detection Only	✓	×	×	0.5368(±0.0197)
A2: Detection + Clipping	✓	✓	×	0.1342(±0.0134)
A3: Full LSTM Framework	✓	✓	✓	0.0827(±0.0141)

Note: ✓ indicates that the corresponding component is enabled, whereas × indicates that it is disabled.

**Table 4:** Performance comparison of defense methods under the Continuous Attack scenario. Values are reported as mean (±standard deviation) over 20 random seeds.

Continuous Attack			
Method	$V_{\mathrm{sys}}$	J	W
No Defense	0.5368(±0.0197)	0.7753(±0.0065)	0.4720(±0.0090)
Z-Score	0.4618(±0.0161)	0.7969(±0.0065)	0.4485(±0.0090)
Isolation Forest	0.0380(±0.0111)	0.9726(±0.0028)	0.3093(±0.0038)
One-Class SVM	0.2590(±0.0132)	0.9380(±0.0011)	0.3100(±0.0088)
AutoGuard, LSTM-only	0.0827(±0.0141)	0.9701(±0.0045)	0.2569(±0.0075)
**AutoGuard-Hybrid**	0.0380(±0.0111)	0.9726(±0.0028)	0.3093(±0.0038)

Note: Bold values indicate the best or tied-best mean performance for each metric.

**Table 5:** Performance comparison of defense methods under the Adaptive Probing Attack scenario. Values are reported as mean (±standard deviation) over 20 random seeds.

Adaptive Probing Attack			
Method	$V_{\mathrm{sys}}$	J	W
No Defense	0.3402(±0.0083)	0.8682(±0.0039)	0.3634(±0.0085)
Z-Score	0.3103(±0.0177)	0.8972(±0.0073)	0.3379(±0.0102)
Isolation Forest	0.0725(±0.0260)	0.9657(±0.0054)	0.3072(±0.0045)
One-Class SVM	0.2502(±0.0156)	0.9396(±0.0010)	0.3104(±0.0090)
AutoGuard, LSTM-only	0.0650(±0.0136)	0.9638(±0.0045)	0.3072(±0.0074)
**AutoGuard-Hybrid**	0.0525(±0.0148)	0.9691(±0.0037)	0.3072(±0.0038)

Note: Bold values indicate the best or tied-best mean performance for each metric.

**Table 6:** Detection stage activation distribution per attack scenario. Values are reported as mean (±standard deviation) over 20 random seeds, with 200 steps per episode.

Scenario	Stage 1, IF	Stage 2, LSTM	Normal	Warm-Up
Continuous	101.0(±0.9)	9.3(±1.2)	80.7(±1.8)	9.0
Adaptive Probing	72.9(±13.3)	29.4(±10.7)	88.7(±4.9)	9.0

## Data Availability

The data presented in this study are available on request from the corresponding author.
